# Osmotic Demyelination Syndrome in a Normonatremic Patient Under Treatment With Proton Pump Inhibitors

**DOI:** 10.7759/cureus.44472

**Published:** 2023-08-31

**Authors:** Lidia Romero Calvo, Maria J Garcia-Blanco, Francisco Valenzuela, Javier Álvarez Granda

**Affiliations:** 1 Internal Medicine, Hospital Central de la Defensa Gomez Ulla, Madrid, ESP; 2 Medicine, Universidad de Alcalá, Alcalá de Henares, ESP; 3 Neurology, Hospital Central de la Defensa Gomez Ulla, Madrid, ESP

**Keywords:** seizures, osmotic demyelination syndrome (ods), omeprazole, hypokalemia, hypomagnesemia

## Abstract

A 66-year-old woman was admitted to the emergency department with diarrhea, nausea, and vomiting as well as low-grade fever. She was initially treated with ciprofloxacin and metronidazole with symptomatic improvement and was discharged. One week later, she returned to the emergency department for gait instability, dizziness, and vomiting and had a witnessed generalized tonic-clonic seizure in the hospital. During both admissions, the presence of ionic alterations such as severe hypomagnesemia, hypophosphatemia, and hypokalemia stood out, while sodium levels remained normal. Among her antecedents, she had a hiatal hernia and had been receiving treatment with omeprazole for years.

## Introduction

Proton pump inhibitors (PPIs) are widely prescribed among the general population but are not exempt from adverse events such as hypomagnesemia, a life-threatening condition that was first related to chronic use of PPIs in 2006 [1-6].

Mutations in genes coding for TRPM6/7 channels lower the absorption of magnesium in the small intestine and decrease the threshold for hypomagnesemia. TRPM6/7 channels can be inhibited by many factors including long-term treatment with PPIs [7-10]. Severe hypomagnesemia can lead to other electrolyte imbalances, such as hypokalemia, hypocalcemia, or hypophosphatemia [9].

Osmotic demyelination syndrome (ODS) is traditionally associated with rapid correction of hyponatremia or hyperglycemia but has also been associated with other electrolytic disorders [11].

## Case presentation

A 66-year-old woman was first admitted to a hospital with a seven-day history of diarrhea, nausea, vomiting, and low-grade fever. She had a previous history of hypertension, a well-controlled type 2 diabetes with hemoglobin A1c (HbA1c) 6.6% and no target-organ damage, hiatus hernia, irritable bowel disease (IBS), and osteoarthritis. Therefore, she was on lisinopril, metformin, tramadol, and omeprazole.

Laboratory findings performed on admission showed an elevated white blood cell (WBC) count, a discrete increase in C-reactive protein (CRP) levels (Table 1), and severe hypomagnesemia, with moderate hypophosphatemia and hypokalemia (Table 2). Both blood and stool cultures were negative. She received treatment with intravenous magnesium for three days and empiric antibiotherapy with metronidazole plus ciprofloxacin for five days. After one week, her symptoms resolved, and she was discharged with no modification to her usual treatment. At the time of discharge, her electrolytes were within normal limits. During hospitalization, a computed tomography (CT) scan abdomen was performed, which showed gastric sheath fold thickening that was interpreted as secondary to the acute gastroenteritis.

**Table 1:** Laboratory test results WBC: White blood cell; CRP: C-reactive protein; LDH: Lactate dehydrogenase; CSF: Cerebrospinal fluid; CPK: Creatine phosphokinase; PCR: Polymerase chain reaction. * Tonic-clonic seizure.

-	Test results						Reference values
-	Day 0	Day 7	Day 15*	Day 19	Day 23	Day 28	-
WBC	16.05	7.63	16.76	9.52	9.2	5,4	4–10 x10^3^/uL
Neutrophil count	12.9	4.37	13.51	6.70	6.63	3.7	1.8–7 x10^3^/uL
CRP	2.23	0.36	0.54	0.48	0.36	0.3	0.01–0.5 mg/dL
Procalcitonin	0.23	0.2	0.04	0.1	0.05	-	<0.5 ng/mL
LDH	296	190	254	178	138	-	135–214 U/L
Fibrinogen	669	308	701	655	550	-	150–450 mg/dL
CPK	-	-	1241	780	250	46	<170 U/L

**Table 2:** Summary of electrolytic disbalances AP: After presentation; FE Mg: Fractional excretion of magnesium. * Tonic-clonic seizure. ** Omeprazole stopped. *** Magnesium supplementation stopped.

During treatment with omeprazole						After omeprazole withdrawal						
-	Day 0	Day 7	Day 15*	Day 19	Day 23**	Day 28	1 month AP	2 months AP***	4 months AP	6 months AP	10 months AP	Reference values
Magnesium	0.61	1.7	0.26	0.75	1.3	1.41	2.02	1.71	1.9	1.88	1.8	1.7–2.55 mg/dL
Sodium	141	139	141	143	145	145	142	143	142	143	140	135–145 mmol/L
Potassium	2.8	4.7	2.5	2.9	3	3	4.4	4.6	4.1	4.6	4.7	3.5–4.5 mmol/L
Phosphate	1.9	3	1.2	2.2	3.1	2.9	3.7	4.2	4	3.7	3.5	2.5–4.5 mmol/L
Clorum	103	102	103	102	102	101	104	105	104	104	105	90–110 mmol/L
Glucose	126	133	168	110	120	120	99	118	113	102	97	76–110 mg/dL
Total proteins	-	-	7.3	9.1	9.3	8.5	6.2	-	-	-	-	6.4–8.7 g/dL
Albumin	4.11	3.53	2.63	-	-	-	3.63	3.47	3.67	3.85	3.76	3–5.5 g/dL
Ionic calcium	-	-	0.76	-	-	-	-	-	-	-	-	1–1.3 mmol/L
Total calcium	-	-	4.9	5.1	6	7.5	9.7	10.2	10.9	10.6	10.1	8–10.4 mg/dL
FE Mg	-	-	-	2.3	-	-	-	-	-	-	-	<3%

One week later, the patient returned to the emergency department due to new persistent dizziness, vomiting without oral tolerance, asthenia, and gait instability. She maintained hemodynamic stability, except for tachycardia at 116 bpm, with no fever. On physical examination, she was found to have alternating nystagmus, cranial right nerve VI palsy, and an increased gait support base. Laboratory findings at this moment showed an elevated WBC count (Table 1) and low magnesium and potassium levels (Table 2). She was admitted to the hospital to pursue further tests on these neurological alterations.

A few hours after admission, she had a tonic-clonic seizure that was aborted with diazepam and levetiracetam. Magnesium sulfate was administered in infusion and was withdrawn two days later. A cranial CT scan showed no evidence of stroke, hemorrhage, or intracranial lesions. Urgent electroencephalogram (EEG) showed no evidence of epileptiform activity. Also, a lumbar puncture (LP) was performed for CSF culture and virus detection on suspicion of central nervous system infection. Ampicillin, cotrimoxazole, and intravenous acyclovir were started empirically and stopped after microbiological tests were negative (Table 1). Pulses of methylprednisolone and immunoglobulins were empirically infused after discontinuation of antibiotics as autoimmune meningoencephalitis was suspected. She kept the treatment for five days, with no improvement in her clinical situation. Onconeuronal antibodies also tested negative (Table 1).

**Figure 1:**
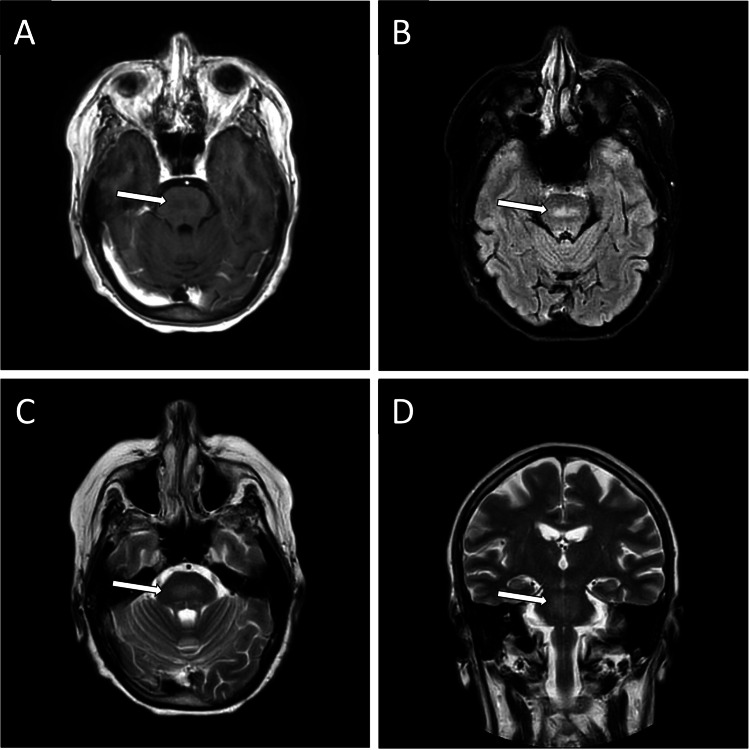
Cranial MRI (A) Central pontine T1W hypointense lesion, (B) and (C) FLAIR and T2W sequences showing central pontine hyperintense areas, and (D) T2W coronal sequence with pontine hyperintensity. T1W: T1-weighted image; FLAIR: Fluid-attenuated inversion recovery; T2W: T2-weighted image.

Magnetic resonance image (MRI) showed age-related white matter changes and highlighted a pontine lesion that measured 8 x 20 x 16 mm. This lesion was hyperintense in T2-weighed sequences and hypointense in T1-weighted sequences, without diffusion restriction or enhancement after contrast administration (Figure 1). These findings were suggestive of ODS.

Since admission, severe hypomagnesemia, hypophosphatemia, hypokalemia, and hypocalcemia were noted, whereas sodium levels were within normal. Endovenous replacement of magnesium, potassium, calcium, and phosphate was required since the patient had another tonic-clonic seizure (Table 2).

An extensive study of these alterations was performed, ruling out urinary and digestive malabsorption as well as digestive or neuroendocrine tumors. Therefore, hypomagnesemia was related to chronic intake of omeprazole. Omeprazole was stopped, and the patient needed regular supplementation of magnesium, calcium, potassium, and phosphorus. Calcium, phosphorus, and potassium supplements could be removed eight days after omeprazole was withdrawn, maintaining magnesium supplements (200 mg, three times a day) at discharge (Table 2). At that moment, the patient no longer presented nystagmus nor VI cranial nerve palsy but maintained gait instability.

**Figure 2:**
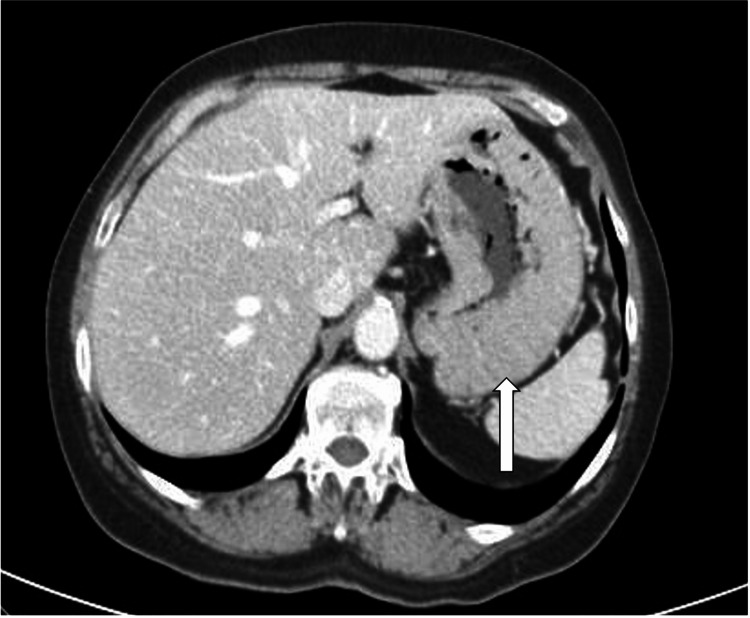
Abdominal CT scan showing gastric wall thickening

A CT body scan was also performed to rule out solid organ neoplasia, highlighting persistent diffuse thickening of the walls of the gastric fundus (Figure 2). Thus, the patient underwent an endoscopic study. The anatomopathological result showed mild chronic gastritis, so treatment with famotidine 20 mg a day was started.

In subsequent reviews in clinics, magnesium levels have remained within the normal range, being able to reduce the dose of oral magnesium progressively until its suspension, two months after its initiation.

To date, the patient is doing well, she no longer has nystagmus or paresis, although gait instability persists. She needs to walk with a cane but maintains independence in her daily activities.

## Discussion

Magnesium is an intracellular cation. Only 1% of the body's magnesium is found in plasma, and of this, 40% is bound to proteins, so plasma magnesium levels are not always representative of the total amount of magnesium in the body [1,2].

Hypomagnesemia involves plasma magnesium levels below 1.7 mg/dL. It can be a life-threatening condition, resulting in neurological and cardiovascular damage and electrolyte disturbances, such as hypokalemia, hypophosphatemia, and hypocalcemia [3]. Neurological manifestations secondary to severe hypomagnesemia occur due to increased neuronal wall excitability, and tetany, seizures, and coma have been described [3,4].

Magnesium absorption occurs at the intestinal and renal levels by active and passive mechanisms [3]. Passive transport of magnesium takes place by diffusion in the small intestine and the distal convoluted tubule of the kidney in a concentration-dependent manner. Active magnesium transport involves transient receptor potential melastatin 6 and 7 (TRPM6/7) receptors found in the cecum and large intestine as well as the distal convoluted tubule at the kidney [3,4].

The association between omeprazole and hypomagnesemia was first reported in 2006 [1] and is now known to be a class effect [5-9]. The proposed mechanism for PPI-induced hypomagnesemia involves TRPM6/7. PPIs decrease the expression of TRMP6/7 channels through alterations in intestinal lumen pH. The activity of these channels at the renal level is not affected. Cases of PPI-induced hypomagnesemia usually occur in patients who have been taking PPIs for a prolonged period of time [9,10].

ODS, classically known as central pontine/extrapontine myelinolysis, occurs in patients who have experienced rapid hyponatremia correction [12]. However, it has also been described in patients with rapid correction of hyperglycemia or ionic imbalances, such as hypokalemia, in anorexic women suffering from refeeding syndrome [12]. The proposed pathophysiologic mechanism is a rapid change in the body's osmostat [13-15], so any sudden change in the osmostat could be a potential causative factor in producing an ODS.

In our case, it was following acute gastroenteritis when the electrolytic imbalances became evident, among which hypomagnesemia predominated. Our patient presented with neurological manifestations after electrolytic replacement during the first admission. These alterations could be attributed to hypomagnesemia, with evidence of pontine lesions on imaging. The ionic disturbances could not be properly corrected despite supplementation until omeprazole was stopped, a treatment that the patient had been taking for more than 15 years. Sodium levels remained normal during both admissions.

## Conclusions

To our knowledge, this is the first case of ODS due to hypomagnesemia in the context of chronic intake of PPIs and acute gastroenteritis. However, a causal relationship between the drug and ODS cannot be established. Clinicians should maintain a high clinical awareness of the potential side effects of prolonged PPI use and closely monitor magnesemia.

Prolonged treatment with PPIs should be suspected in the presence of ionic alterations of unclear cause. A genetic study of TRPM 6/7 could be useful in patients who have a greater predisposition to brain damage and who need treatment with PPIs. PPI-associated ionic disturbances can have fatal consequences. Further studies are needed in this direction if more cases are reported in the future.
